# Robotic‐assisted laparoscopic radical prostatectomy for treatment of a newly identified lesion revealed no viable cells in the previously treated area with microwave focal therapy

**DOI:** 10.1002/iju5.12733

**Published:** 2024-05-14

**Authors:** Fumio Tsukuda, Toshihiro Shimizu, Kiichi Hagiwara, Yoshiyuki Kawano, Noboru Sakamoto, Shingo Itagaki, Yutaka Horiguchi, Shoji Koga, Osamu Ukimura

**Affiliations:** ^1^ Department of Urology Edogawa Hospital Tokyo Japan; ^2^ Department of Pathology Edogawa Hospital Tokyo Japan; ^3^ Department of Urology, Graduate School of Medical Science Kyoto Prefectural University of Medicine Kyoto Japan

**Keywords:** microwave focal therapy, mpMRI, MR/US fusion‐guided targeted biopsy, prostate cancer, RARP

## Abstract

**Introduction:**

Histological outcome of the targeted focal therapy is in principle confirmed by targeted needle biopsy from the treated area in clinical trial. Herein, we report a rare case in which the MFT was followed by RARP.

**Case presentation:**

A 68‐year‐old man with PSA 9.6 ng/mL and PI‐RADS 4 lesion in the right transition zone on multi‐parametric MRI underwent MR/ultrasound fusion‐guided targeted biopsy, which revealed grade‐group 1 cancer. Targeted focal therapy with microwave ablation was performed, resulting in disappearance of the PI‐RADS 4 lesion at post‐operative 4 months. However, PSA rose to 11.5 ng/mL, and a new PI‐RADS 4 lesion, was identified in the left peripheral zone. RARP was performed to reveal new grade‐group 3 cancer, and no viable cells in the previously treated area with MFT.

**Conclusion:**

RARP was safely performed even after MFT and proved the pathological complete response of microwave ablation.

**Abbreviations & Acronyms**
ADC: apparent diffusion coefficient
DWI: diffusion‐weighted image
FT: focal therapy
GG: grade group
HIFU: high‐intensity‐focused ultrasound
IIEF‐5: International Index of Erectile Function‐5
IPSS: International prostate symptom score
MFT: microwave focal therapy
mpMRI: multi‐parametric magnetic resonance imaging
PI‐RADS: Prostate Imaging—Reporting and Data System
PSA: prostate‐specific antigen
PZ: peripheral zone
RARP: robotic‐assisted laparoscopic radical prostatectomy
RP: radical prostatectomy
TRUS: transrectal ultrasound
TZ: transition zone

**Keynote message**

RARP was safely performed even after microwave focal therapy and proved the complete response in the treated lesion by previous microwave ablation.

## Introduction

Although the standard treatment for localized prostate cancer is RP, some of those cases could be controlled by FT.^1^ FT has proven efficacy and safety in prospective clinical trials and is recommended by clinical guidelines.^2^, ^3^, ^4^, ^5^, ^6^, ^7^, ^8^ In our experience, the patients, who had a recurred cancer in treated area or a new cancer lesion identified in untreated area, RP or repeated FT is available as salvage therapy option. Here, we report the rare case, in which PSA did not drop even after successful MFT, underwent RARP because of a newly identified cancer in untreated prostate.

## Case presentation

**Fig. 1:**
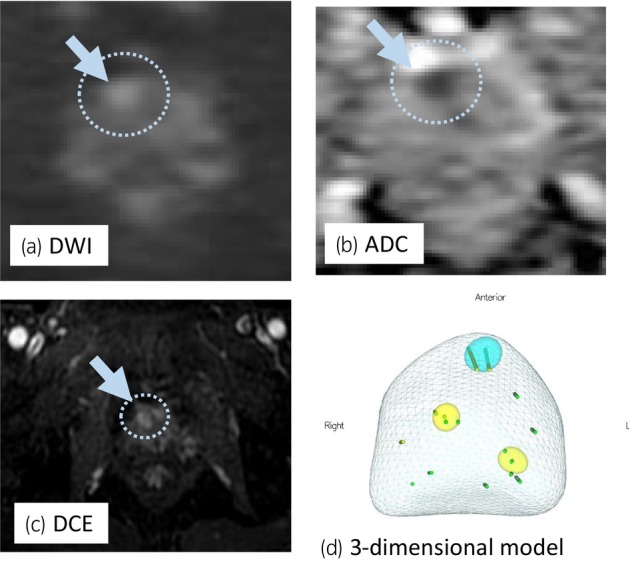
Targeted lesion of FT in pre‐operative MRI. Multiparametric MRI revealed PIRADS‐category 4 lesion in right anterior TZ with 13 mm in diameter (a–c; blue arrow and circle). MR/US fusion targeted biopsy found GG 1 (blue lesion, in d). No cancer were seen in other targeted biopsies (yellow lesions, in d) and any systematic biopsies.

**Fig. 2:**
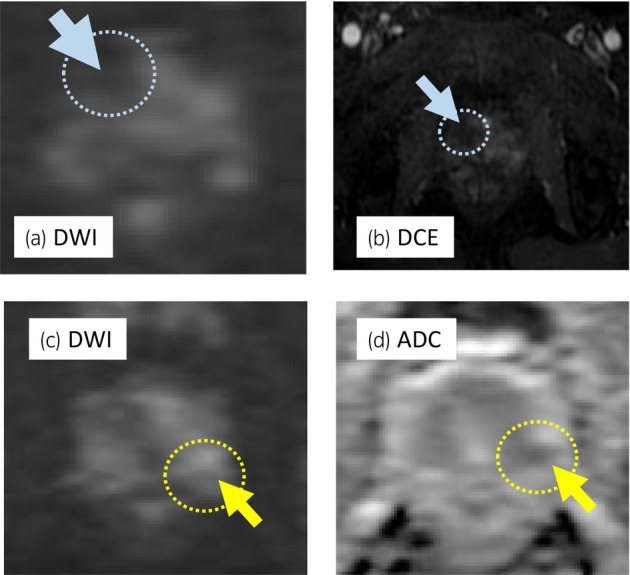
Surveillance MRI at 4 months after FT. The treated targeted lesion of focal therapy was normalized in DWI, and no enhancement in DCE (blue arrow with circle in a, b). MRI at 4 months after focal therapy revealed a new lesion of PIRADS‐category 4 in the left PZ (yellow arrow and circle in c, d).

**Fig. 3:**
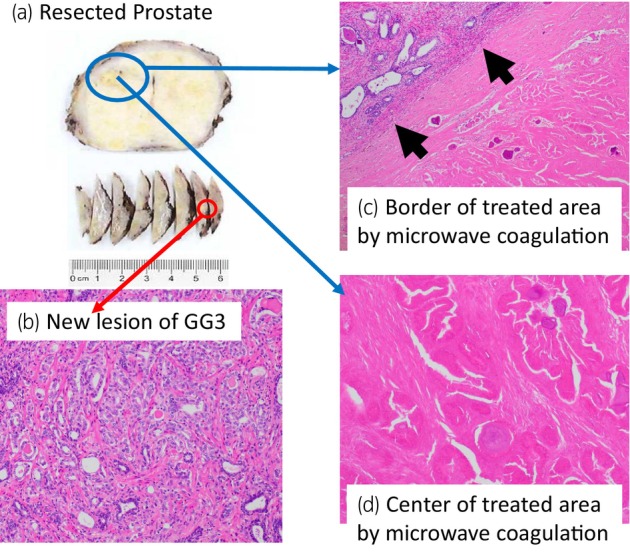
Pathological analysis of robot‐assisted RP. In the whole mount section of resected prostate (a), blue circle was shown the treated area of microwave coagulation in TZ and the new cancer lesion in the apical prostate PZ was shown as red circle. GG 3 prostate cancer was seen in the new lesion (b, 10×). The treated area by microwave coagulation had clear border (black arrows, in c) and totally coagulative necrosis was observed with no viable cancer‐cells (in d, 20×).

A 68‐year‐old man with PSA of 9.66 ng/mL, who had PI‐RADS 4 lesion with 13 mm in diameter in the right anterior TZ on mpMRI (Fig. 1a–c), underwent transperineal MR/US fusion‐guided targeted needle biopsy (TRINITY™; Koelis, Meylan, France). Pathological diagnosis revealed GG 1 cancer in three cores of the targeted lesion in right TZ. No cancers were diagnosed in all the other targeted and systematic biopsy cores (Fig. 1d). The patient underwent a total of six sessions of MFT (MicrotaseTM; Alfresa Pharma Corporation, Osaka, Japan) to the biopsy‐proven targeted TZ cancer lesion. Each ablation session was set as 30 W for 60 s to coagulate an ellipsoid area with major axis of 20 mm and minor axis of 12 mm. The operation time was 60 min with no intraoperative complications. 4 months after the MFT, the ablated cancer lesion disappeared in MRI (Fig. 2a,b), but the PSA rose to 11.59 ng/mL after treatment. MRI revealed a new PI‐RADS 4 lesion with 7 mm in diameter in the left lateral posterior PZ in untreated area (Fig. 2c,d). Then, RARP were underwent in the usual manner without any intraoperative complications. Total operation time was 240 min and blood loss was 30 mL. Histopathological examination of the resected specimen revealed localized GG 3 (G4 pattern 80%) cancer in the left apical PZ (negative surgical margin, Fig. 3a,b). No viable cells were found in the treated area with previous MFT, where had coagulative necrosis with a clear border to the adjacent normal prostate gland (Fig. 3a,c,d). PSA decreased under detectable limits 3 months after RARP.

## Discussion

The goal of FT is to less invasively preserve urinary and sexual function with cancer control as compared to invasive standard whole‐gland therapy.^5^, ^8^ Boku *et al*. revealed the safety and efficacy of microwave tissue coagulation for prostate cancer by a non‐clinical study and exploratory clinical trial.^9^ Oderda *et al*. reported early functional outcomes of 11 patients with single MRI‐visible prostate cancer who underwent transperineal targeted microwave ablation. No significant changes in IPSS and IIEF‐5 scores were seen.^10^ Barry Delongchamps *et al*. reported performing MFT on 10 patients of low‐ to intermediate‐risk cancer with a visible index tumor on MRI and MRI showed total necrosis in 8 patients and cancer were not seen in four patients by re‐biopsy at 6 months.^11^

Marconi *et al*. reported that most of the multi‐center cohort study of 82 patients received HIFU or cryotherapy, the continence rate, defined as using no pads, was 83.1%. They mentioned salvage RARP was safe and has arguably excellent urinary continence outcomes.^12^ Cathcart *et al*. reported toxicity of salvage RARP after FT was low, with similar urinary and sexual function outcomes to primary cases of RP.^13^ However, no report was found on salvage RARP after MFT.

In this case, 4 months after MFT, biochemical and radiological new lesion in the non‐treated area of the MFT were observed. PSMA‐PET was not done because it was not common in Japan. This was the only single case in which PSA never decreased and rose from the value of pre‐operative PSA after MFT among the experience of 36 cases in our institution.

Although we did not have selection criteria regarding recurrence after MFT, the patient chose to perform RARP because he had voiding difficulty and desired radical treatment. In addition, the patient agreed to undergo RARP at this point with further elevated PSA without re‐biopsy before RARP. The reason to undergo no re‐biopsy before RARP was since the patient, who had already been diagnosed as prostate cancer, desired to avoid the re‐biopsy toward the highly suspicious for new MRI‐visible cancer lesion with having further elevated PSA. Also, the patient had understood that another clinically significant cancer can be identified in the follow‐up after MFT as sampling error at the time of the initial diagnosed biopsy that was performed as combination of both systematic and image‐guided targeted techniques.

Although, we had confirmed no cancer in the left PZ target (Fig. 1d) at the initial diagnostic biopsy, GG3 prostate cancer with 5 mm in diameter was seen in resected specimen of RARP. This was rare but a current clinical limitation of under‐diagnosis as MRI‐based selection failure and could be an unavoidable case in current recommended biopsy combined 10–12 cores systematic biopsy with targeted biopsy. Salvage RARP was safely performed without any complications and the radicality for cancer was secured even after MFT. Histopathological examination confirmed complete response in coagulative ablation of cancer lesion and technically the targeted area of MFT had been precisely ablated as planned. Herein, we first proved the histological complete response in the entire target cancer area, as one of the most important issues of MFT.

## Conclusion

This is the first report that RARP was safely performed even after MFT and proved the complete ablation of the targeted cancer lesion by previous targeted microwave ablation. We conclude that MFT can be the promising surgical options for localized prostate cancer.

## Author contributions

Fumio Tsukuda: Methodology; writing – original draft; writing – review and editing. Toshihiro Shimizu: Validation. Kiichi Hagiwara: Validation. Yoshiyuki Kawano: Validation. Noboru Sakamoto: Validation. Shingo Itagaki: Data curation. Yutaka Horiguchi: Validation; writing – review and editing. Shoji Koga: Supervision. Osamu Ukimura: Conceptualization; supervision; writing – review and editing.

## Conflict of interest

The authors declare conflict of interest for Alfresa Pharma K.K. about support our clinical trial.

## Approval of the research protocol by an Institutional Reviewer Board

Not applicable.

## Informed consent

Written informed consent was obtained.

## Registry and the Registration No. of the study/trial

Not applicable.
